# Association between clinic-level quality of care and patient-level outcomes in multiple sclerosis

**DOI:** 10.1177/13524585231181578

**Published:** 2023-06-30

**Authors:** Anna H He, Ali Manouchehrinia, Anna Glaser, Olga Ciccarelli, Helmut Butzkueven, Jan Hillert, Kyla Anne McKay

**Affiliations:** Department of Clinical Neuroscience, Karolinska Institute, Stockholm, Sweden/Centre for Molecular Medicine, Karolinska University Hospital, Stockholm, Sweden; Department of Clinical Neuroscience, Karolinska Institute, Stockholm, Sweden/Centre for Molecular Medicine, Karolinska University Hospital, Stockholm, Sweden; Department of Clinical Neuroscience, Karolinska Institute, Stockholm, Sweden; Queen Square MS Centre, Department of Neuroinflammation, UCL Queen Square Institute of Neurology, Faculty of Brain Sciences, University College London, London, UK; Department of Neuroscience, Central Clinical School, Monash University, Melbourne, VIC, Australia; Department of Clinical Neuroscience, Karolinska Institute, Stockholm, Sweden; Department of Clinical Neuroscience, Karolinska Institute, Stockholm, Sweden/Centre for Molecular Medicine, Karolinska University Hospital, Stockholm, Sweden

**Keywords:** Multiple sclerosis, quality of care, evidence-based healthcare, patient-reported outcome measures

## Abstract

**Background:**

Multiple sclerosis (MS) quality of care guidelines are consensus-based. The effectiveness of the recommendations is unknown.

**Objective:**

To determine whether clinic-level quality of care affects clinical and patient-reported outcomes.

**Methods:**

This nationwide observational cohort study included patients with adult-onset MS in the Swedish MS registry with disease onset 2005–2015. Clinic-level quality of care was measured by four indicators: visit density, magnetic resonance imaging (MRI) density, mean time to commencement of disease-modifying therapy, and data completeness. Outcomes were Expanded Disability Status Scale (EDSS) and patient-reported symptoms measured by the Multiple Sclerosis Impact Scale (MSIS-29). Analyses were adjusted for individual patient characteristics and disease-modifying therapy exposure.

**Results:**

In relapsing MS, all quality indicators benefitted EDSS and physical symptoms. Faster treatment, frequent visits, and higher data completeness benefitted psychological symptoms. After controlling for all indicators and individual treatment exposures, faster treatment remained independently associated with lower EDSS (−0.06, 95% confidence interval (CI): −0.01, −0.10) and more frequent visits were associated with milder physical symptoms (MSIS-29 physical score: −16.2%, 95% CI: −1.8%, −29.5%). Clinic-level quality of care did not affect any outcomes in progressive-onset disease.

**Conclusion:**

Certain quality of care indicators correlated to disability and patient-reported outcomes in relapse-onset but not progressive-onset disease. Future guidelines should consider recommendations specific to disease course.

## Introduction

Recent national and international practice guidelines (The Swedish Multiple Sclerosis Association^1^ and other national^2^ and international consortia^3–6^) have sought to standardise the care received by persons living with multiple sclerosis (MS). These quality indicators were developed through expert consensus opinion (level 5 evidence), but empirical evidence for their effectiveness is lacking. The health resource expenditure required to meet these quality indicators must be justified, particularly given the expense to payers, competing demands on healthcare providers, and perceived inferiority of care in more resource-limited settings where such standards are not feasibly met.^7^ Specifically, the recommendations should have a demonstrable and positive effect on patient outcomes. Yet, to date, these outcomes have not been evaluated.

Sweden has a universal healthcare system with theoretically equitable access to care regardless of demography or ability to pay. Over 60 neurology clinics are responsible for provision of care to persons living with MS in Sweden, each of which record observational clinical data and patient-reported outcomes via the Swedish MS registry (SMSReg^8^).

Using the SMSReg, our aim was to investigate the effectiveness of current national quality-of-care (QOC) recommendations in improving patient outcomes. These consensus-based guidelines were first developed by the Swedish MS Society in 2009, with regular revisions thereafter. The current edition, released in 2016, has been harmonised with the federal Department of Social Welfare guidelines for MS care.^2^

We hypothesised that better performance on quality indicators may lead to more favourable clinical outcomes in relapse-onset MS, and that this is likely mediated by clinics’ ability to rapidly initiate and optimise treatment. Secondarily, we hypothesised that better QOC may lead to improvements in patient-reported outcomes in all subtypes of MS.

## Materials and methods

### Study design, participants and setting

This was an observational cohort study using data from the Swedish MS registry, which contains prospectively recorded individual patient information from neurology clinics across Sweden (2001–present). The registry captures approximately 80% of all prevalent cases of MS in the population.^8^ Participation is voluntary and all patients provide informed consent for their data to be used for clinical and research purposes. Data were available until 31 December 2019.

Study participants were individuals with incident adult-onset (⩾18 years of age at first symptom) MS, with onset between 1 January 2005 and 31 December 2015 (to exclude the initial years of the registry which were more likely to be missing incident cases and data, and to provide 4 years of follow-up to the end date). Only clinics with ⩾20 study-eligible patients registered in the SMSreg at the date of data extraction were included, to ensure only active clinics were included. For inclusion in the outcomes analyses, patients were required to have at least one recorded outcome (Expanded Disability Status Scale (EDSS) or Multiple Sclerosis Impact Scale (MSIS-29)).

### Exposure

The exposure of interest was the quality of care provided at patients’ clinics during the calendar year of their disease onset, as measured by the following four domains:

1. Mean visit density,^1,3^ calculated as total number of MS patient visits, divided by the number of MS patients, in that clinic during the specified calendar year.
2. Mean magnetic resonance imaging (MRI) density,^6,9,10^ calculated as the number of MRI scans for MS patients, divided by the number of MS patients, in that clinic during the specified calendar year.
3. Data completeness,^1,3,4^ calculated as the number of incident cases in the calendar year with complete baseline data, divided by the clinic’s total number of incident cases in that calendar year. Complete baseline data included date of birth, sex, dates of symptom onset and diagnosis, and at least 1 EDSS recorded within 2 years of diagnosis.
4. Mean treatment delay,^1,3,4,11–15^ calculated as a clinic’s mean time between symptom onset to first disease modifying therapy (DMT) recorded, for all patients in that clinic who had symptom onset within that calendar year.

### Outcomes

Outcomes included (1) EDSS and (2) MSIS-29. The EDSS^16,17^ is a clinical measure of disability, determined by history and neurological examination. It is an ordinal scale from 0 (no disability) to 10 (death), with the smallest increment being 0.5, except one increment from 0 to 1.0. The MSIS-29^18^ is a disease-specific patient-reported outcome measure. It is a 29-item questionnaire used to report the presence and severity of physical (20 items) and psychological (9 items) symptoms of MS that patients experienced in the previous 2 weeks. The physical and psychological scores are converted to percentage scores out of 100, with higher values indicating more severe symptoms. Each of these outcomes were repeatedly measured approximately annually.

Patients were followed from their MS symptom onset until their most recent outcome measurement recorded (EDSS or MSIS-29).

### Statistical analysis

All statistical analyses were performed using R version 4.1.3.^19^ Descriptive summary statistics were calculated for each QOC domain for each clinic per calendar year between 2005 and 2015 and presented graphically.

Repeated-measures patient outcomes were assessed using mixed models with two-level clustering at the patient and clinic level, to account for dependency of outcomes from the same patient and the same clinic, respectively. EDSS scores were modelled using a linear mixed model. MSIS-29 physical and psychological subscale scores were modelled using a generalised linear mixed model with a log-link gamma function to account for the gamma-distributed response variable. Zeroes in this response variable were handled by assigning them the lowest possible positive score in each subscale. To adjust for individual disease severity, the following patient-level covariates were included: age at disease onset, sex, disease duration at the time of each outcome measure, and number of relapses recorded in the first 2 years of disease^20^ (for relapse-onset patients). All models were stratified by disease course (relapse-onset and progressive-onset).

We first modelled each QOC indicator separately, adjusting for the above-mentioned patient-level covariates. Additionally, for relapse-onset patients, we included individual patients’ treatment with high- and modest-efficacy DMTs, to assess whether the effectiveness of the quality indicators was independent of intensity and duration of treatment.^21–24^ Finally, after excluding multicollinearity by ensuring the variance inflation factor (VIF) of all model covariates (again including treatment with disease-modifying therapies) was less than two, we included all indicators in one model to assess their effect, independent of one another.

Individual patients’ treatment exposure was modelled as the proportion of disease time (between disease onset and time of each outcome measure) treated with high- and modest-efficacy therapies. High-efficacy treatments included rituximab, ocrelizumab, mitoxantrone, alemtuzumab, natalizumab and haematopoietic stem cell transplant. Modest-efficacy therapies included interferon-beta, glatiramer acetate, fingolimod, dimethyl fumarate, teriflunomide, cladribine and siponimod.

Ethical approval was granted by the Stockholm County Ethical Review Board (approval number: 2017/1378-31).

## Results

### Descriptive analyses

**Figure 1.**
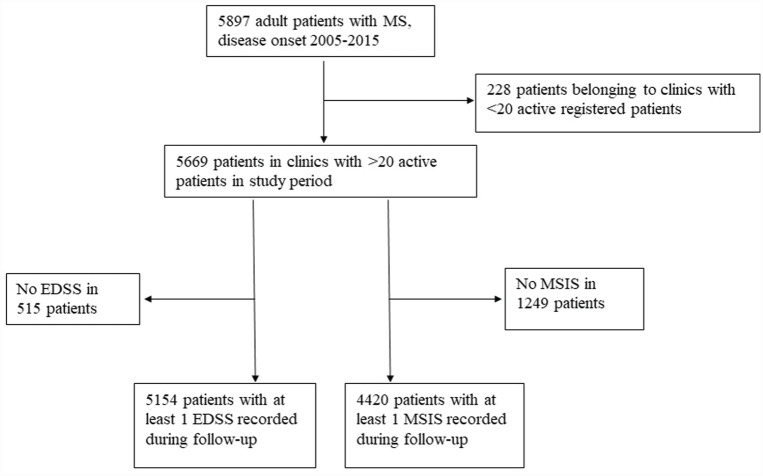
CONSORT chart of patient selection.

**Figure 2.**
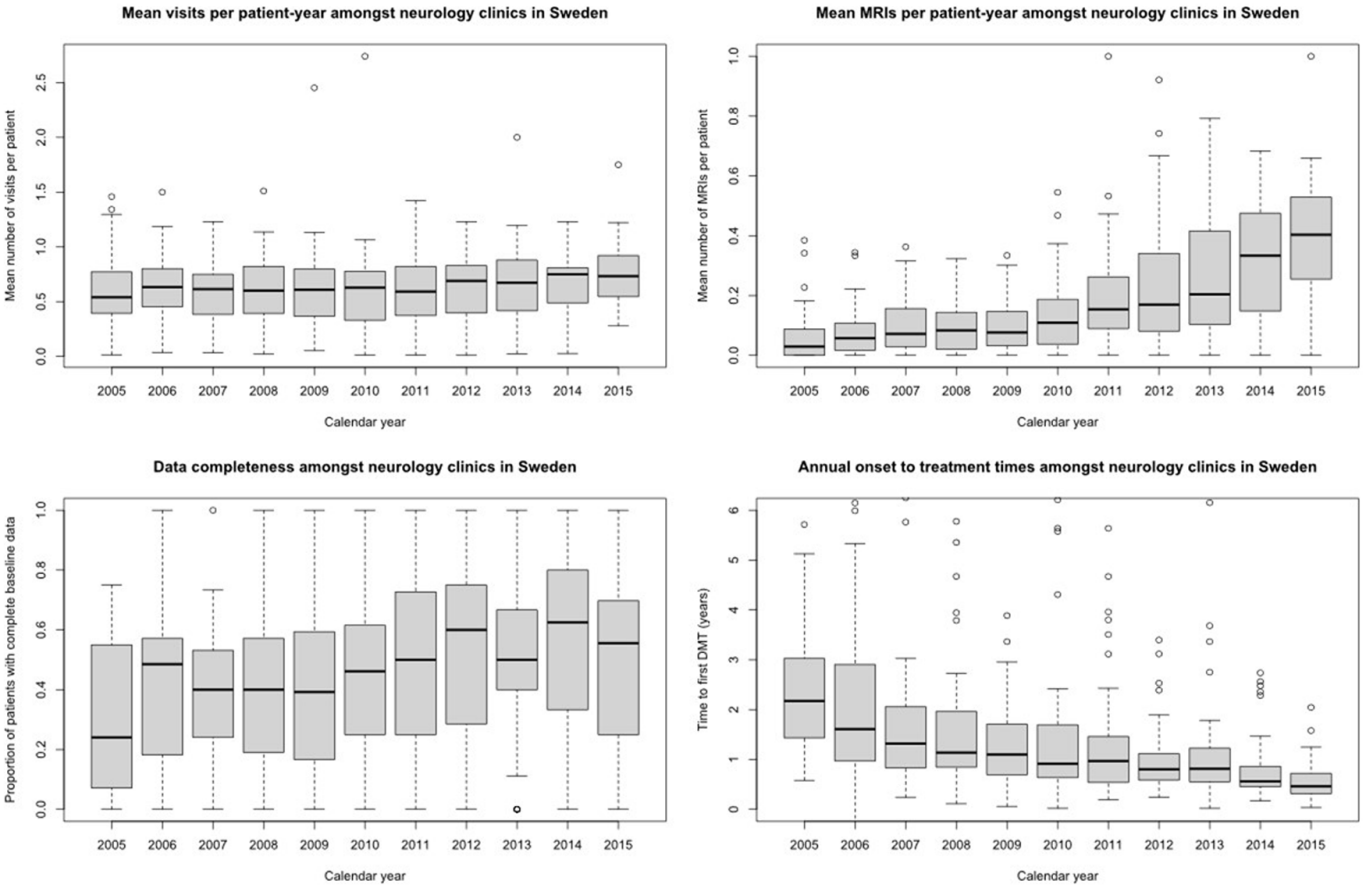
Annual performance in four quality indicators among neurology clinics in Sweden, 2005–2015.

We identified 5669 patients from 48 eligible clinics in the SMSReg with clinical onset of MS between January 2005 and December 2015 (Figure 1). Year-by-year summary statistics of the 48 clinics’ performance on QOC indicators are provided in Figure 2 and Supplemental Table S1.

### Association between QOC and EDSS

The EDSS analysis included 5154 (Figure 1) patients (69.1% female) with a median (inter-quartile range (IQR)) follow-up of 8.16 (5.59, 10.91) years (Table 1).

**Table 1.** Baseline characteristics of patients stratified by disease course at onset.

	EDSS analysis		MSIS analysis	
	Relapsing	Progressive	Relapsing	Progressive
*n*	4802	352	4215	205
Age at onset (mean (SD))	34.50 (9.77)	45.40 (8.85)	33.79 (9.43)	42.98 (9.33)
Males (%)	1419 (29.6)	172 (48.9)	1251 (29.7)	99 (48.3)
Calendar year of onset (median (IQR))	2010 (2007, 2013)	2009 (2007, 2012)	2010 (2008, 2013)	2010 (2008, 2012)
Treatment delay, days (median (IQR))	320 (127, 915)		321 (124, 935)	
*N* relapses in first 2 years (median (IQR)) Mean (SD)	1.0 (0.0, 1.0) 1.02 (1.17)	0.0 (0.0, 0.0) 0.16 (0.50)	1.0 (0.0, 2.0) 1.04 (1.18)	0.0 (0.0, 0.0) 0.20 (0.59)
Mean (SD) number of EDSS recorded per person	7.33 (4.88)	5.49 (3.90)	5.44	3.27

Among 4802 patients with relapse-onset MS, all four quality indicators were associated with subsequent EDSS scores. EDSS was lower in patients attending clinics with faster treatment times (0.12 points lower per 1-year reduction in treatment delay, 95% confidence interval (CI): 0.08, 0.17), higher visit density (0.2 points lower per one additional visit per patient-year, 95% CI: 0.04, 0.36), higher MRI density (0.58 points lower per 1 additional MRI per patient-year, 95% CI: 0.34, 0.83), and higher rate of data completeness (0.04 points lower for per 10% increase in baseline data completeness, 95% CI: 0.02, 0.06).

The summary of estimates for QOC indicators is provided in Table 2. Similar results were observed when individual patients’ treatment exposures were included in the analyses (Supplemental Table S2).

**Table 2.** Estimated effect of quality-of-care indicators on EDSS, indicators modelled individually.

Quality indicator	Relapse-onset				Progressive-onset			
	Est.	LL	UL	*p*	Est.	LL	UL	*p*
Clinic visit density^a^	−0.200	−0.363	−0.036	0.017	0.571	−0.211	1.353	0.155
Clinic MRI density^b^	−0.584	−0.827	−0.340	<0.001	0.095	−1.166	1.356	0.883
Clinic data completeness^c^	−0.042	−0.064	−0.020	<0.001	0.092	−0.017	0.201	0.101
Clinic treatment delay^d^	0.123	0.079	0.168	<0.001				

Each model was adjusted for: age at onset, sex, disease duration at outcome measurement. Patient ID and clinic ID were modelled as random intercepts.

a Estimates represent change in EDSS per one additional visit per patient-year.

b Estimates represent change in EDSS per one additional MRI per patient-year.

c Estimates represent change in EDSS per 10% increase in clinics’ baseline data completeness.

d Estimates represent change in EDSS per additional year of mean treatment delay.

When all QOC indicators were modelled together with individual patient DMT exposure, individual patients’ proportion of time on treatment with high- and modest-efficacy DMTs had the largest effect on EDSS in relapse-onset MS. A shorter time to treatment continued to show a beneficial effect on EDSS, but the effect size was markedly attenuated (0.06 points higher for every year of treatment delay at the clinic level (95% CI: 0.01, 0.10; Table 3). No other quality indicator remained independently associated with EDSS. There was low multicollinearity between variables (VIF < 2 for all comparisons).

**Table 3.** Estimated effect of quality-of-care indicators on EDSS: combined analysis with all indicators, adjusting for sex, age, disease duration, relapses and proportion of time on modest- and high-efficacy disease-modifying therapies.

Variable	Relapse-onset				Progressive-onset			
	Est.	LL	UL	*p*	Est.	LL	UL	*p*
EDSS at intercept	1.07	0.87	1.27	<0.001	1.19	0.23	2.14	0.015
Male	0.14	0.06	0.23	0.001	0.24	−0.14	0.62	0.215
Age (years over 18 at onset)	0.03	0.03	0.04	<0.001	0.03	0.01	0.05	0.006
Disease duration (years)	0.06	0.05	0.06	<0.001	0.22	0.20	0.23	0.000
Relapses in first 2 years	0.10	0.07	0.14	<0.001				
Proportion of disease time treated with high-efficacy therapy	−0.48	−0.54	−0.43	<0.001				
Proportion of disease time treated with modest-efficacy therapy	−0.31	−0.34	−0.27	<0.001				
Clinic visit density^a^	−0.09	−0.27	0.08	0.300	0.53	−0.32	1.38	0.224
Clinic MRI density^b^	−0.15	−0.45	0.14	0.301	−0.47	−1.85	0.91	0.506
Clinic data completeness^c^	−0.02	−0.04	0.01	0.162	0.08	−0.03	0.20	0.166
Clinic treatment delay^d^	0.06	0.01	0.10	0.027				

a Estimates represent change in EDSS per one additional visit per patient-year.

b Estimates represent change in EDSS per one additional MRI per patient-year.

c Estimates represent change in EDSS per 10% increase in clinics’ baseline data completeness.

d Estimates represent change in EDSS per additional year of mean treatment delay.

Among 352 patients with progressive-onset MS, no quality indicator was associated with an altered EDSS (Tables 2 and 3).

### Association between QOC and MSIS-29

The MSIS-29 analysis included 4420 patients (69.5% female) with a median (IQR) follow-up of 8.02 (5.51, 10.75) years.

Among 4215 patients with relapse-onset MS, all QOC indicators were significantly associated with subsequent physical symptoms (Table 4). Patients attending clinics with longer time to DMT initiation reported 13.4% greater physical symptoms for every year of delay (95% CI: 8.2, 18.9), higher visit frequency was associated with 19.4% lower symptom burden (95% CI: 7.7%, 29.7%), higher MRI frequency was associated with 35.4% lower symptom burden (95% CI: 18.8%, 48.6%), and higher data completeness with 3.8% lower symptom burden (95% CI: 1.9%, 5.7%). When individual treatment exposures were included in the analyses, the association between the quality indicators and physical symptoms was similar; however, the estimated effect of MRI frequency was no longer statistically significant (see Supplemental Table S3).

**Table 4.** Estimated effect of quality-of-care indicators on MSIS-29 score, indicators modelled individually.

Quality indicator	Relapse-onset				Progressive-onset			
	Est.	LL	UL	*p*	Est.	LL	UL	*p*
Physical subscore								
Clinic visit density^a^	0.806	0.703	0.923	0.002	1.248	0.831	1.874	0.286
Clinic MRI density^b^	0.646	0.514	0.812	<0.001	0.846	0.302	2.370	0.750
Clinic data completeness^c^	0.962	0.943	0.981	<0.001	0.976	0.904	1.054	0.542
Clinic treatment delay^d^	1.134	1.082	1.189	<0.001				
Psychological subscore								
Clinic visit density^a^	0.908	0.811	1.017	0.096	1.248	0.831	1.874	0.286
Clinic MRI density^b^	0.868	0.718	1.050	0.145	0.846	0.302	2.370	0.750
Clinic data completeness^c^	0.990	0.990	0.991	<0.001	0.976	0.904	1.054	0.542
Clinic treatment delay^d^	1.066	1.025	1.108	0.001				

Each model was adjusted for: Age at onset, sex, disease duration at outcome measurement. Patient ID and clinic ID were modelled as random intercepts.

Estimates are multiplicative of reference value; estimates <1 indicate lower symptom burden, >1 indicate higher symptom burden.

a Estimates represent change in MSIS-29 symptom score per one additional visit per patient-year.

b Estimates represent change in MSIS-29 symptom score per one additional MRI per patient-year.

c Estimates represent change in MSIS-29 symptom score per 10% increase in clinics’ baseline data completeness.

d Estimates represent change in MSIS-29 symptom score per additional year of mean treatment delay.

Slower mean time to treatment was associated with worse psychological symptoms (6.6% increase, 95% CI: 2.5%, 10.8%), while other QOC indicators showed minimal effect (Table 4).

No quality indicator remained associated with psychological symptoms after adjusting for treatment exposure (see Supplemental Table S4).

Treatment with high- and modest-efficacy therapies had the strongest effect size in both physical and psychological symptoms in relapse-onset MS (Table 5). After adjusting for both DMT exposure and other QOC indicators, higher visit density remained significantly associated with a 16% reduction in patient-reported physical symptoms (95% CI: 2%, 29%). No other QOC indicator was independently associated with subsequent physical symptoms. None of the QOC indicators independently affected psychological symptoms.

**Table 5.** Estimated effect of quality-of-care indicators on MSIS-29 score: combined analysis with all indicators, adjusting for sex, age, disease duration, relapses and proportion of time on modest- and high-efficacy disease-modifying therapies.

Variable	Relapse-onset				Progressive-onset			
	Est.	LL	UL	*p*	Est.	LL	UL	*p*
Physical subscore								
MSIS physical score at intercept	11.14	9.29	13.35	<0.001	17.98	9.55	33.85	< 0.001
Male	0.83	0.76	0.91	<0.001	0.97	0.72	1.31	0.868
Age (years over 18 at onset)	1.02	1.02	1.03	<0.001	1.02	1.00	1.03	0.043
Disease duration (years)	1.06	1.02	1.10	0.004	1.03	1.00	1.05	0.018
Relapses in first 2 years	1.01	1.01	1.02	<0.001				
Proportion of disease treated with high-efficacy therapy	0.53	0.50	0.56	<0.001				
Proportion of disease treated with modest-efficacy therapy	0.65	0.62	0.68	<0.001				
Clinic visit density^a^	0.84	0.71	0.98	0.029	1.39	0.86	2.25	0.174
Clinic MRI density^b^	1.13	0.84	1.52	0.416	0.79	0.26	2.46	0.689
Clinic data completeness^c^	0.98	0.95	1.00	0.070	0.96	0.88	1.05	0.376
Clinic treatment delay^d^	1.05	1.00	1.11	0.058				
Psychological subscore								
MSIS psychological score at intercept	30.30	26.50	34.65	<0.001	34.09	19.56	59.39	< 0.001
Male	0.83	0.77	0.88	<0.001	0.84	0.64	1.11	0.220
Age (years over 18 at onset)	1.00	1.00	1.00	0.847	1.00	0.99	1.02	0.643
Disease duration (years)	0.99	0.98	0.99	<0.001	1.00	0.98	1.02	0.969
Relapses in first 2 years	1.03	1.00	1.06	0.041				
Proportion of disease treated with high-efficacy therapy	0.60	0.57	0.63	<0.001				
Proportion of disease treated with modest-efficacy therapy	0.69	0.66	0.72	<0.001				
Clinic visit density^a^	0.90	0.80	1.02	0.094	0.99	0.66	1.49	0.952
Clinic MRI density^b^	1.17	0.94	1.46	0.156	1.33	0.51	3.46	0.565
Clinic data completeness^c^	1.00	0.98	1.02	0.788	0.96	0.89	1.03	0.281
Clinic treatment delay^d^	1.01	0.97	1.05	0.763				

ID and clinic ID were modelled as random intercepts.

Estimates are multiplicative of reference value; estimates <1 indicate lower symptom burden, >1 indicate higher symptom burden.

a Estimates represent change in MSIS-29 symptom score per one additional visit per patient-year.

b Estimates represent change in MSIS-29 symptom score per one additional MRI per patient-year.

c Estimates represent change in MSIS-29 symptom score per 10% increase in clinics’ baseline data completeness.

d Estimates represent change in MSIS-29 symptom score per additional year of mean treatment delay.

There was low multicollinearity between variables in either model (VIF < 2 for all).

Among 205 patients with progressive-onset MS, no quality indicator was associated with change in any patient-reported symptoms (Tables 4 and 5).

## Discussion

This study explored the relationship between clinic-level quality of care and clinical disability and patient-reported symptoms of MS. We demonstrated that quality indicators were associated with clinical and patient-reported outcomes in relapsing MS, and that this relationship persisted to a large extent when individual-level disease-modifying therapy was included in the analysis. Notwithstanding, only faster treatment times and higher visit densities were independently associated with lower subsequent disability and physical symptoms when all quality indicators and treatments were included in one model. This aligns with previous studies that demonstrated treatment to be more effective if given early^25^ and provides additional evidence that frequent visits can help with symptom management.

No quality indicator had an independently beneficial effect on psychological symptoms, nor did any indicator have any measurable benefit on longer-term outcomes for progressive-onset patients.

These findings reflect current MS management paradigms that primarily optimise for clinical disability in relapse-onset disease, namely rapid treatment initiation and close monitoring for breakthrough disease. While MRI is necessary for initial diagnosis, the role of MRI activity in making treatment decisions during follow-up is debated.^26,27^ This study demonstrates that those clinics with high MRI frequency indeed achieved better clinical and patient-reported outcomes, and that this was driven by other factors such as treatment decisions. The same was seen for clinics’ data completeness, which has no independent effect on patient outcomes but may facilitate better patient management. The purpose of systematic data entry has primarily been for benchmarking and research, and its utility in these areas is not under question by this study.

No quality indicator demonstrated benefit in progressive-onset disease, perhaps reflecting a lack of evidence-based interventions for this group, and thereby the failure of current guidelines to address the specific needs of this group. Another consideration regarding the negative findings in progressive-onset patients is that the estimated effect size and variance of each quality indicator were likely diluted in our study due to averaging of these at a clinic level. Using clinic-level metrics, rather than the QOC received by patients individually, attempts to minimise indication bias while still capturing the heterogeneity of care provided at different clinics. However, due to loss of data granularity in an already small subgroup of progressive patients, it cannot be excluded that some quality indicators had a true effect on outcomes but were unable to be detected due to limited statistical power.

Outside of treatment guidelines, the importance of other aspects of quality of care is less well-studied. Of the quality indicators included in this study, only ‘onset to treatment time’ had existing evidence in relapsing MS.^11–14^ While international committees are highly concurrent regarding other indicators such as clinic frequency and MRI frequency, the evidence for their direct benefit is lacking. One observational study showed that higher visit frequency was associated with poorer prognosis, but this was likely due to indication bias.^28^ The necessity of frequent MRI scans and appointments may be self-evident in those for whom treatment safety and efficacy monitoring is mandatory, but less evident for those not eligible for active treatment. Indeed, this study confirms that these interventions are beneficial for relapse-onset, but not progressive-onset disease. Future guidelines should provide targeted recommendations for patients’ disease course rather than blanket recommendations. Future research in quality indicators may further stratify recommendations on the basis of other patient characteristics such as age, sex, symptom constellation or markers of disease severity.

This study only included four QOC parameters. Other guideline recommendations that are likely to play a significant role in patient outcomes were not included, due to lack of data or inability to be measured, such as the quality of the individual patient–doctor relationship,^29^ the extent to which decision-making was shared and person-centred,^3,30^ and the availability of care on an as-needed rather than per-schedule basis.^1,3^

As demonstrated in the descriptive analysis of QOC in Sweden over time, our study captured a wide range of QOC performances but are still subject to boundary effects within the studied clinics. The results are applicable to universal health care contexts where the variance in QOC received by persons with MS may be small, but further study is needed in other contexts. Finally, this study assumes that QOC is independent of socioeconomic status in Sweden. While this is true in theory regarding a person’s ability to access care, socioeconomic status may be associated with healthcare-seeking behaviours^31–34^ as well as outcomes. The relationship between higher QOC and improved long-term outcomes may be partly explained by socioeconomic status. Future studies should examine the relationship between socioeconomic status, quality of care and MS-specific outcomes.

## Conclusion

Quality of care for persons with MS varies between neurology clinics. Quality guideline recommendations primarily benefit relapse-onset MS, and the benefit is imparted partly by individual-level DMT exposure. Future guidelines should target recommendations by disease course, according to evidence for their effectiveness.

## Supplemental Material

sj-docx-1-msj-10.1177_13524585231181578 – Supplemental material for Association between clinic-level quality of care and patient-level outcomes in multiple sclerosis Click here for additional data file. Supplemental material, sj-docx-1-msj-10.1177_13524585231181578 for Association between clinic-level quality of care and patient-level outcomes in multiple sclerosis by Anna H He, Ali Manouchehrinia, Anna Glaser, Olga Ciccarelli, Helmut Butzkueven, Jan Hillert and Kyla Anne McKay in Multiple Sclerosis Journal
