# Supplementary material for: Association between clinic-level quality of care and patient-level outcomes in multiple sclerosis
Source: Mult Scler. 2023 Jun 30;29(9):1126–35. doi: 10.1177/13524585231181578 (PMC10413789; doi:10.1177/13524585231181578)
Supplement: sj-docx-1-msj-10.1177_13524585231181578 – Supplemental material for Association between clinic-level quality of care and patient-level outcomes in multiple sclerosis [file sj-docx-1-msj-10.1177_13524585231181578.docx]

| **Calendar year** | **2005** | **2006** | **2007** | **2008** | **2009** | **2010** | **2011** | **2012** | **2013** | **2014** | **2015** |
| --- | --- | --- | --- | --- | --- | --- | --- | --- | --- | --- | --- |
| Delay to diagnosis (days), mean (SD) | 601.82 (580.11) | 536.95 (342.03) | 604.72 (661.10) | 534.72 (530.07) | 427.76 (345.40) | 353.07 (354.01) | 399.51 (473.27) | 359.61 (285.57) | 299.23 (284.56) | 289.50 (256.80) | 192.66 (182.87) |
| Delay to DMT (days), mean (SD) | 1031.71 (867.23) | 771.20 (594.80) | 717.80 (726.72) | 631.77 (602.82) | 483.25 (306.70) | 554.53 (632.59) | 522.40 (543.53) | 422.72 (406.87) | 398.11 (392.45) | 304.27 (242.60) | 180.88 (213.86) |
| Visits per patient (mean (SD)) | 0.59 (0.33) | 0.64 (0.31) | 0.61 (0.28) | 0.62 (0.31) | 0.63 (0.38) | 0.62 (0.41) | 0.62 (0.32) | 0.64 (0.32) | 0.65 (0.37) | 0.68 (0.26) | 0.74 (0.27) |
| MRIs per patient (mean (SD)) | 0.06 (0.09) | 0.08 (0.08) | 0.10 (0.09) | 0.11 (0.10) | 0.10 (0.09) | 0.14 (0.13) | 0.20 (0.18) | 0.23 (0.20) | 0.26 (0.21) | 0.33 (0.20) | 0.39 (0.21) |
| Proportion with complete baseline data (mean (SD)) | 0.31 (0.25) | 0.40 (0.28) | 0.38 (0.23) | 0.40 (0.28) | 0.39 (0.26) | 0.44 (0.28) | 0.50 (0.30) | 0.53 (0.29) | 0.53 (0.27) | 0.57 (0.31) | 0.50 (0.29) |
| N new patients (median [IQR]) | 6.5 [3.0, 14.25] | 7.0 [3.0, 14.25] | 9.0 [4.0, 15.00] | 8.5 [3.75, 15.5] | 7.5 [3.0, 13.0] | 8.0 [3.0, 15.25] | 8.0 [4.0, 17.25] | 7.0 [4.0, 17.0] | 6.0 [2.75, 18.25] | 7.0 [3.0, 13.25] | 7.0 [4.0, 13.0] |
| N total patients (median [IQR]) | 41.5 [15.25, 116.0] | 73.0 [20.0, 130.5] | 94.5 [32.75, 164.25] | 123.0 [44.0, 209.5] | 134.0 [54.0, 252.25] | 141.0 [57.75, 276.5] | 153.5 [63.5, 300.0] | 171.0 [81.75, 328.75] | 183.0 [86.25, 349.0] | 198.0 [96.0, 373.75] | 212.0 [104.5, 399.75] |

**Supplementary Table 1: summary of neurology clinics’ annual performance on 4 quality domains from 2005 to 2015**

**Supplementary material: Effect of clinic-level quality of care on longitudinal patient outcomes in relapsing MS, adjusted for individual patient treatment exposure**

| **Table S2: Estimated effect of QoC indicators on EDSS: adjusted for treatment exposure** | | | | |
| --- | --- | --- | --- | --- |
|  | Est | LL | UL | p |
| Time to treatment | 0.08 | 0.03 | 0.12 | 0.001 |
| Clinic visit density | -0.18 | -0.34 | -0.01 | 0.035 |
| Clinic MRI density | -0.39 | -0.63 | -0.14 | 0.002 |
| Clinic data completeness | -0.03 | -0.06 | -0.01 | 0.003 |

**Each model was adjusted for:** Age at onset, sex, disease duration at outcome measurement, proportion of disease time treated with high-efficacy therapy, proportion of disease time treated with lower-efficacy therapy. Patient ID and clinic ID were modelled as random intercepts.

| **Table S3: Estimated effect of QoC indicators on physical symptoms (MSIS-29 physical subscale): adjusted for treatment exposure** | | | | |
| --- | --- | --- | --- | --- |
| Quality indicator | Est | LL | UL | p |
| Time to treatment | 1.06 | 1.01 | 1.12 | 0.016 |
| Clinic visit density | 0.80 | 0.69 | 0.92 | 0.003 |
| Clinic MRI density | 0.80 | 0.61 | 1.03 | 0.089 |
| Clinic data completeness | 0.97 | 0.95 | 0.99 | 0.003 |

**Each model was adjusted for:** Age at onset, sex, disease duration at outcome measurement, proportion of disease time treated with high-efficacy therapy, proportion of disease time treated with lower-efficacy therapy. Patient ID and clinic ID were modelled as random intercepts.

NB: estimates are multiplicative of reference; values above 1 indicate higher symptom burden

| **Table S4: Estimated effect of QoC indicators on psychological symptoms (MSIS-29 psychological subscale): adjusted for treatment exposure** | | | | |
| --- | --- | --- | --- | --- |
| Quality indicator | Est | LL | UL | p |
| Time to treatment | 1.00 | 0.97 | 1.04 | 0.857 |
| Clinic visit density | 0.92 | 0.83 | 1.02 | 0.128 |
| Clinic MRI density | 1.06 | 0.89 | 1.27 | 0.512 |
| Clinic data completeness | 1.00 | 0.98 | 1.01 | 0.815 |

**Each model was adjusted for:** Age at onset, sex, disease duration at outcome measurement, proportion of disease time treated with high-efficacy therapy, proportion of disease time treated with lower-efficacy therapy. Patient ID and clinic ID were modelled as random intercepts.

NB: estimates are multiplicative of reference; values above 1 indicate higher symptom burden
